# Exploring the experience of chronic pain among female Survival Sex Workers: a qualitative study

**DOI:** 10.1186/s12875-015-0395-6

**Published:** 2015-12-21

**Authors:** Caroline Allen, Alka Murphy, Sheri Kiselbach, Stephanie VandenBerg, Ellen Wiebe

**Affiliations:** PACE Society, British Columbia, Vancouver, Canada; Department of Emergency Medicine, Cumming School of Medicine, University of Calgary, Calgary, Canada; Department of Family Practice, University of British Columbia, Vancouver, Canada

**Keywords:** Sex worker, Chronic pain, Drug use, Pain management

## Abstract

**Background:**

The prevalence of self-identified chronic pain in Canadian adults is approximately one in five people. Marginalization and addictions have been shown to complicate chronic pain in vulnerable populations. This study aimed to understand the experience of chronic pain among female Survival Sex Workers in Vancouver's downtown eastside (DTES).

**Methods:**

This study used an exploratory qualitative analysis with in-depth, semi-structured interviews. Members of PACE Society who self-identified as a current or former Survival Sex Worker and who had a chronic pain experience known to PACE support workers were invited to participate. Interviews were conducted, audio recorded and transcribed. The investigators met to read the transcripts and discuss emerging themes. The process continued until no new themes were observed.

**Results:**

Participants ranged in age from 42 to 56 years old and all self- identified as females and Survival Sex Workers. Eleven of thirteen interviews were analyzed for themes. Drug use for pain management, both prescribed and illicit, was the most important theme. Poverty, the need to continue working and the lack of stable housing were barriers to adequately addressing the source of chronic pain. Participants felt judged for living in the downtown eastside, being a drug user and/or being Aboriginal and only two participants had been referred to a pain specialist. All participants were involved in support networks made up of other Sex Workers and all spoke of a sense of community and survival.

**Conclusions:**

Our study emphasizes the complex nature of chronic pain and addictions among a uniquely marginalized population. The study is unique in that it contributes the perspectives of a traditionally “hard-to-reach” population and demonstrates that Sex Workers should not only participate in but should lead development and implementation of research and programs for managing chronic pain in the setting of addiction.

## Background

The prevalence of self identified chronic pain in Canadian adults, operationally defined as intense pain that is experienced several times per week for more than six months, has been estimated to be 18.9 % [1]. Chronic pain has many implications for individuals and the larger community, manifested by physical functional impairment, psychological stress, workplace absenteeism, and decreased community engagement resulting in an overall decrease in quality of life. Chronic pain carries with it a significant expense, costing the Canadian healthcare system over 6 billion dollars per year in direct medical costs and lost workplace productivity [2–4]. Past paradigms of chronic pain have understood chronic pain to be a symptom of an injury or disease, concluding that treatment of the cause should cure the pain. New understandings of chronic pain now challenge us to conceptualize chronic pain as a sum that is greater than its parts, that is, pain that persists well after the original insult has healed, leaving long-lasting abnormal sensory findings secondary to changes in how peripheral nerves, spinal cord and brain react to non-noxious stimuli [3].

Based on this paradigm, a key component to understanding chronic pain, what initiates, perpetuates and alleviates it, means placing the individual pain experience within the larger social, political and economic climate [5]. For our purposes, this means understanding the politics of gender, federal law, and the social factors influencing these areas. Previous studies have documented how gender places women at higher risk for chronic pain then their male counterparts [6, 7]. From a political point of view, current federal laws surrounding communication and prohibition of indoor spaces occupied for the purpose of prostitution criminalize “primarily vulnerable women facing various difficulties including poverty, homelessness and drug dependency” [6]*.* Survival sex work in Vancouver’s downtown east side is primarily street based and therefore subject to this federal criminal law.

Social and economic factors such as homelessness and low-income have been shown to increase perceptions of chronic pain, with Hwang et al’s study identifying a disproportionate prevalence of chronic pain among homeless populations compared to the national average [7–11]. Disparities in chronic pain management by primary care providers (PCPs) towards populations with a history of active or remote substance misuse were further compounded by provider stereotypes. Specifically, studies showed that PCPs impressions of a patient’s opiate use was discordant from patient self-reports and that non-white patients and patients with concurrent substance use disorders were less likely to be treated with opiates for chronic pain management [12–14].

In Vancouver’s downtown eastside (DTES), where our study took place, chronic pain has been identified in community based projects featuring the work of marginalized women. The Maka Project and VANDU (Vancouver Area Network of Drug Users) Women Care study provided insight into health negotiation for women who use illicit drugs in the DTES, specifically focused on primary health care experiences [15, 16]. Their research included data on pain experiences and attempts to seek treatment for pain. They found that 28 % of the women interviewed reported chronic pain problems, with 45.7 % of women using drugs to cope with pain. A further 39.6 % had reported “trouble getting medication when needed and being denied pain medication when they requested it” [16–20] No study has taken a dedicated look at the experience of chronic pain in female Survival Sex Workers.

Survival Sex Work is defined as: “the lack of opportunity to consistently refuse to work in dangerous circumstances” [21]. This lack of opportunity may be due to poverty, homelessness, physical/mental health, addiction, and stigma. These issues are made more complex by larger factors such as a history of childhood neglect, abuse and/or sexual abuse, the legacy of colonialism, residential schools, exploitation, predatory violence and the criminalization of negotiation (Section 213 of the Canadian Criminal Code).

Providing Alternatives, Counseling and Education Society (PACE Society) is a non-profit grassroots organization operating in Vancouver’s DTES that is by, with and for Sex Workers. The organization was established in 1994 by former Sex Workers and their allies who recognized that some services for Sex Workers were best provided by Sex Workers. PACE Society provides frontline, confidential one-to-one support, accompaniment and advocacy for a wide range of self-identified needs. Their programs include street outreach and violence prevention education for Survival Sex Workers located in and outside of the DTES. Approximately 80 % of the Sex Workers accessing PACE Society are Survival Sex Workers [21].

The purpose of this research was two-fold. Firstly, it sought to address the gap in social sciences and health literature on the experience of chronic pain in female Survival Sex Workers, a topic that was identified by the study population as an issue they wanted to explore. Secondly the research aimed to bear witness to each individual story, giving voice to a traditionally marginalized and hard-to-reach community.

## Methods

### Research design

This study used an exploratory qualitative analysis with in-depth interviews to explore chronic pain in female Survival Sex Workers. All five authors participated in the design of the study. Research Ethics was granted by the University of British Columbia Research Ethics Boards. Participant signed consent was obtained after reviewing verbally, for those who were illiterate, and on paper the risks, benefits, implications and alternatives to participating in the study. All study participants provided signed consent. Semi-structured interviews used the following structured domains and allowed for open-ended questioning: “Experiences and expressions of chronic pain [in words or in art]”; “Therapies/Treatments”; and “Medical Community”. Interviews were completed by one researcher (SV), transcribed and analyzed by two researchers (SV, EW) during the three months that the interviews were being conducted and used to inform subsequent interviews based on emerging themes. Significant statements were grouped according to common themes using manual analysis techniques generating initial codes by hand and combining codes into overarching themes. PACE Society staff were consulted as content experts to ensure accurate interpretation and application of themes and theoretical perspectives. Suggestions from the staff resulted in new themes and the transcripts were again reviewed until saturation of these themes was achieved, the point where no new information was obtained from subsequent interviews. Transcripts of all interviews conducted are stored on the primary author’s password protected computer and are available upon written request, in keeping with data availability requirements.

Methodological rigor was achieved by verification, validation and ensuring credibility. Verification was accomplished through a thorough literature search, identifying researcher bias, and achieving data saturation [23]. Validation was achieved by triangulation of data between participants and staff experience and re-occurring thematic analysis by the research team [24]. By identifying participants with lived experiences of chronic pain, accurately representing their experiences and triangulating data, we sought to achieve credibility of the research [25].

### Participants

PACE Society support staff invited women already known to them through one-to-one support relationships, and who had previously disclosed their challenges around chronic pain and would be willing to participate in hour-long interviews to take part in the study. Inclusion criteria consisted of: age greater than 18 years, current or former Survival Sex Worker who self-identified as female, Member of PACE Society and who experienced chronic pain. Participants were excluded if they did not speak English fluently or if they were actively psychotic.

A purposeful sample of fifteen eligible participants was identified by staff and met inclusion criteria. Two participants did not attend their scheduled interview with the remaining thirteen participants, aged 42 – 56 years old (mean age 50) giving informed consent to participate in qualitative interviews about their chronic pain experience. Two consenting participants demonstrated active psychosis and their interview data was not included in the analysis. A total of eleven interviews were analyzed. Six women identified as Aboriginal^1^ while the remainder were of Caucasian descent. Interviews lasted between 30 minutes to 1 hour 27 minutes.

### Consent to publish

All participants provided their signed consent to have their anonymous data individualized and included in this publication.

### Data gathering

Interviews were conducted over a three-month period of time (October – December 2012) and participants were compensated for their time. To ensure safety and accessibility, the interviews took place in the PACE Society office, located in Vancouver’s DTES. A staff support worker was available to support the participant before, during and after the interview. Adhering to the TriCouncil Policy Statement, informed consent was obtained after verbal explanation of the study and assurance of confidentiality. All interviews were performed by the same interviewer (SV) and used previously agreed upon open-ended questions to further explore topics. A sample size was estimated by previous phenomenological studies and interviews were conducted until theme saturation occurred [24]. All interviews were audio-recorded and transcribed verbatim by someone different than the interviewer and were reviewed for accuracy.

## Results

Five key themes emerged from the women’s experiences of chronic pain. They were: communication, cures and addictions, barriers, stressors and support systems.

### Theme one: communication

#### Understanding chronic pain

The language used to describe chronic pain was diverse, ranging from words like*“pitch black with lightening bolts in it*” (Participant 7), *“little spurts, like surprises”* (Participant 11) and *“causes lots of anger and depression”* (Participant 2). Women distinguished chronic pain from acute pain by comparing it to pain experiences that had a finite endpoint. A sense of defeat and lack of agency as a consequence of persistent, debilitating pain was a common theme. One of the women related the recurrence of abuse in her life to her physical experience of pain and emotional feelings of worthlessness.

*And you can’t go without having pain. It just doesn’t happen. You know. But the pain that I’ve had through my life has been non-stop. From beatings and neglect and beat up a lot on myself, a lot where, you know, you don’t think, um, you’re good enough for anything. So you live in that pain of feeling that worthlessness, that void.* (Participant 1)

#### Communicating with others

The theme of *“not being heard”* (Participant 3) revolved around barriers in communication between medical professionals and the women themselves when it came to chronic pain. Women repeatedly described their experiences of being *“ignored”* (Participant 1)*,* of feeling *“rejected”* (Participant 4). Others felt like they and their physician were speaking different languages. *“I don’t know these doctors, like they have big words you can’t even understand”* (Participant 1). These interactions resulted in women not wanting to access medical care even in emergency situations. *“Ever since my hospital experiences, I’m traumatized now. You try and get me into a hospital, I ain’t going until I’m almost on my death bed”* (Participant 4).

### Theme two: “Cures”

Drug use was the most prevalent and most discussed theme among all participants. All participants were polydrug users. Substance use was described in three ways: self-medicating with substances not prescribed by a healthcare provider including alcohol, marijuana, cocaine, and various opiates; medications that were prescribed by a physician specifically for treatment of chronic pain; and alternative practices such as the practice of smudging. In all cases, the goal of using a substance was to achieve a desired effect, most commonly described as a *“numbing”* (Participant 3) or a *“way of coping”* (Participant 10).

Women described their experience with illicit substances as a way to get *“all my feelings out”* or as a way of not feeling at all.

*“So I went on a binge for about a month. Just drank everyday. And, it didn’t help, but it got all my feelings out”* (Participant 2).

Obtaining certain types of medications was not an issue for some women with family physicians, describing them as *“very accessible”* (Participant 10). Others noted that medications could be bought off the street or off of friends, in the absence of a prescription. *“My doctor, uh, gives me 15. But you just go up to Main and Hastings and you can buy them”* (Participant 3). Another changed her opiates depending on what was available, saying *“if I can’t get heroin I’ll go get dilaudids”* (Participant 11).

Other women, however, encountered significant barriers in accessing pain management strategies, including medications*. “And then having to deal with the, you know, not being able to get anything for pain or, you know, is like, is just like holy fuck. It’s very defeating. Right?”* (Participant 13).

Two women were offered resources through a chronic pain clinic while others were declined and still others *“didn’t even know they existed”* (Participant 11).

On the opposite end of the spectrum were those who stated they didn’t *“need medicine to cure myself sometimes”.* These woman sought pain relief in traditional Native medicines in place of *“white man’s medications”.*

*I’m First Nation and when it’s comes down to medication, I’d rather go to our medicine doctor or just go to my sage.* (Participant 3)

### Theme three: systemic barriers

Women described multiple systemic barriers in managing their chronic pain including judgment and poverty. The most common theme was that of judgment in the form of stigma. They felt judged for being from a certain area,

*You know, people think they’re all scum down here, but you know what, that’s - they didn’t start here. You know, this is just a place they ended up... And some of them don’t know how to get out of it.* (Participant 2)

First Nations women felt discriminated against for having a certain ethnic background. Others felt labeled for having a substance dependence. *“Like once you’re labeled as an addict, like, in – in so many physicians’ eyes, that’s all you are and that’s all you’re out to get”* (Participant 10).

Other common themes that emerged under the topic of barriers were those associated with poverty and the effect of low-income and inadequate housing on managing a chronic condition. Income came primarily from sex work and was largely supplement by disability, welfare and pension payments when unable to work. Prescribed lifestyle changes and therapies for chronic pain were described as inaccessible or unaffordable. One woman recalled, *“I should be going to physio but I can’t afford it. You’re only allowed so many a year and then you have to pay the user fee. Who’s gonna pay that?”* (Participant 12) while others cited acupuncture and massage as being inaccessible due to the cost.

Women expressed frustration over the cost of prescription medications, especially those that were not covered or those who’s coverage was delayed.

*You have to pay 200 dollars for fucking medications. It’s not covered by medical or status. 200 bucks. You can take your fuckin’ meds and shove it up your ass, you’re going to tell me that it’s not covered* (Participant 3)

Housing conditions that were affordable were often inadequate. One woman described, *“fleas in the carpet, mice in the stove, um, cockroaches in the bathroom. Oh it was a horrible place, and I got stuck staying there for 2 years”* (Participant 2).

Women expressed frustration accessing the emergency department as a resource for pain due to stigma as well as frustration with building a relationship with a physician knowing their histories as both a Sex Worker and an addict. One participant felt so rejected that it was easier for her to reject herself, saying,

*I don’t wanna go back through not being listened to again, do you know what I mean there, right?... If you can reject it first before somebody rejects you, it’s easier to shut it down. It’s that wall you put up, right?* (Participant 4)

### Theme four: stressors

All participants experienced adversities in their early years and continued to experience ongoing stressors. Unstable relationships, lack of family support, past and current trauma and the effect of chronic pain on mood were the most commonly described stressors in the lives of the participants. Four women described their previous relationships as outright abandonment, *“After I told him I was pregnant, he just left”* (Participant 2). One woman’s boyfriend had recently committed suicide while another women’s boyfriend was caught *“fooling around”* (Participant 2)*.* Women described an utter lack of family supports. One woman states *“My family’s disowned me 'cause I’m a drug addict”* (Participant 4).

Outside of a lack of supports, all but one of the women described experiencing trauma, from loss through miscarriages to rape, to the emotional trauma of aborting a child conceived from that rape, to leaving home at age thirteen and getting *“thrown in county jail”* (Participant 2) for working the streets, to losing daughters from AIDS. Women described physical beatings by husbands, boyfriends and clients. Many women made the link between the *“stress of having to, you know, deal with my pain”* (Participant 13) and carry the weight of traumatic experiences,

### Theme five: support

Amidst the physical and emotional pain, all of the women described a sense of a surrogate family in the form of counsellors and community in the downtown eastside. Others spoke of counselors at Three Bridges Clinic and within their supportive housing units. One woman spoke of her family physician advocating for her. Women used words like *“on the ball”* (Participant 11) and *“she had compassion for me”* (Participant 4) to described good patient-physician interactions they had experienced, with one woman describing how meaningful it had been that her family physician visited her while she was in the hospital.

Peer-to-peer run programs such as PEERS (Prostitutes Empowerment Education Resource – no longer in operation in Vancouver), WISH Drop-In Centre Society, VANDU (Vancouver Area Network of Drug Users) and PACE Society were described as *“life-saving”* (Participant 8)*.* Women specifically spoke about PACE Society staff and their empowerment and advocacy roles in a peer-to-peer setting.

*“I always felt honored about who I am, you know, and validated and supported and I get more help from them than I do [laughs] with the doctors I go to seek help from.”* (Participant 8)

The idea of strength, of surviving and giving back to the community was a common sentiment. One participant, despite her own *“emotional pain”* had adopted the role of a *“grief and loss counsellor for the last 13 years”* and tries *“to be strong for the community. And for the people down here. And then with my pain”* (Participant 13).

Finally, three of the women spoke about the success of peer-based 12 step programs in managing addictions and extrapolated this idea to include support groups for people with chronic pain.

*That would be really good to see. There is for, you know, cessation groups for smoking and AA [Alcoholics Anonymous] and NA [Narcotics Anonymous] and yeah, like chronic pain support group would be a really good idea.* (Participant 8)

One woman suggested designing, *“a module, you know, like the NA thing or whatever for people, you know, like in my position with chronic pain and addiction”* (Participant 13).

## Discussion

In our study, Survival Sex Workers described complex factors including misrepresentation, poverty, homelessness, physical/mental health, and addiction. Verbalizing what it means to experience chronic pain as well as attempting to communicate this to family, friends and health professionals was a common challenge. Communication with medical professionals was complicated by the phenomenon of assumed drug seeking in the clinical setting. Judgment, polysubstance use, poverty and an overall lack of ability to advocate for oneself and one’s management of chronic pain led to a perception that medical professionals were not listening. Exploring paradigms of chronic pain, addictions and its origins help us to reason why medical professionals have contributed to this perceived neglect. If chronic pain is seen as being temporally related to a painful stimulus that should resolve with treatment of that stimulus, if addiction is seen as a choice and non-compliance viewed as a psychological or biological factor, then we incorrectly focus our energies on changing the patient. Instead, with a chronic pain and addictions paradigm that shifts from a biologic understanding of dyscompliance to an understanding of the social factors, cultural, political and economic factors that contribute to a perceived non-compliance, we can begin to address the multiplicity of factors that have failed this population in the first place and continue to feed a culture of stigma around chronic pain and addictions [26].

Self-medication strategies and polysubstance use blurred the lines of therapy and addiction. Many of the women had insight into their polysubstance use as a coping strategy. A Lancet study published in 2005 confirms alcohol to be the most important drug in the sex-industry, similar to the findings in our research study [27]. From a harm reduction point of view, detailed assessment of both pain experiences and addictions histories present opportunities for physicians to participate in reducing the harms associated with illicit drug use [16]. It also stands to reason that if pain management is a priority for an individual and pain is being undertreated or not treated at all, that a person may return to licit or illicit drugs to “cure” the pain. In their paper, “Universal Precautions”, the authors note that it is possible for addictions and pain to co-exist as medical conditions, for example in the case of neuropathic pain associated with alcoholism. They do note, though, that the case of opiate addiction and chronic pain may be it’s own beast, where the medical treatments for chronic pain, opiates and other medications, may themselves be simultaneously the problem, the solution, or a mixture of the two [26].

Participants sentiments on “occupational stigma” as a barrier to care, associated with being a sex worker, addict or both has been well described in the literature [16–19, 28, 29]. Ahern et al. hypothesize that isolation secondary to stigma further contributes to poor physical and mental health, demonstrated most vividly in a refusal to access healthcare, even when it was badly needed [30]. In addition, woman in this study spoke of stigma as a type of secondary pain and described the challenges of coping with yet another dimension of their experience.

The challenges of successfully managing chronic pain amidst inadequate living conditions echoed previous study findings. One group of researches investigating policies for urban Aboriginal populations has described homelessness as a symptom “of larger problems, including substance abuse, mental health issues, family breakdown, underemployment, low income, and racism” [31]. According to our study’s findings, any one of the factors listed in addition to ongoing challenges in managing chronic pain could be a symptom of homelessness itself. It is hard to determine which comes first. It may not be appropriate to reduce the analysis to one root cause when all of the women described the dynamic interplay of all of the factors that have marginalized them and prevented them from accessing appropriate management of their chronic pain. Of note, Aboriginal women experiencing chronic pain were disproportionately represented in our study. This finding correlates with VANDU Women Care study findings that describe Aboriginal women as being “over-represented among Survival Sex Workers in the DTES … Seventy percent of Sex Trade Workers in the DTES are Aboriginal women and mothers of at least one child” [31–33].

While initial understandings of pain were rooted and expressed in terms of their physical manifestations, participants identified the relationship between their ability to cope with the physical symptoms and the mental and emotional impact of previous violence, neglect and abuse in their lives. This is well supported in studies that have been conducted in Vancouver’s DTES and other North American centres that have found “significant numbers of street-based sex workers have histories of childhood sexual and physical abuse, increasing their susceptibility to mental and emotional problems” [15, 18, 19, 27, 34–37].

Despite feelings of rejection, the women interviewed had found strength in solidarity, in peer-to-peer support groups and empowering partnerships with each other and, rarely, with their physician advocates. The women regarded themselves as their own pain and addictions experts and were frustrated by the lack of acknowledgement and, with some empowering exceptions, the complete rejection of their lived experience.

### Limitations

This study is limited by its design as an exploratory qualitative analysis of a specific participant population who were already accessing a support centre at PACE Society and who experience chronic pain, although a formal diagnosis may not have been previously made. It is not generalizable to other Survival Sex Workers experiencing chronic pain. We took steps to minimize bias and the need for participants to feel like they should tell the researchers what they wanted to hear by partnering closely with staff, interviewing in a familiar and safe place and using open-ended questions. The fact that all participants stayed for more than 15 minute interviews leant support to the idea that chronic pain was something participants wanted to talk about and were not just there for the compensation.

### Future directions

While literature on chronic pain in female Survival Sex Workers is lacking, interventions that use “accessible, acceptable, high-quality, integrated care” have shown reduction in drug use, disease, violence and exploitation [27]. Collective communities such as those found at Sex Worker organizations like WISH and PACE Society reflect community-based empowerment models that have proved effective in improving access to health services in other settings [38–41]. Further study should be made to investigate the role of peer-to-peer group programs in supporting Survival Sex Workers with chronic pain in the setting of addictions. An attempt to quantify a return-on-investment strategy would also be a novel way of evaluating the current cost of interventions specific to this group and what it could save the public healthcare system down the road. The goal of future studies will be to incorporate community based participatory action research methods to allow Survival Sex Workers and other stakeholders to investigate strategies that best meet the needs of Survival Sex Workers with chronic pain and addictions.

## Conclusions

Our study emphasizes the complex nature of chronic pain and addictions among a uniquely marginalized population. Our findings support previous studies that have found it incomplete to talk about chronic pain without discussing the factors that perpetuate, exacerbate, and alleviate the experience of chronic pain in a specific population [5]. Participants identified these factors to be issues around communication, pharmacotherapy and alternative therapies, systemic barriers in the form of stigma and poverty, chronic stressors and the value of peer-to-peer support. This study builds on existing knowledge by suggesting that chronic pain in the setting of addictions and marginalization may be a symptom of more complex concerns associated with polysubstance use, trauma, poverty and stigma, in addition to mental and physical health issues. The findings of our study further support the idea proposed by Gourlay et al’s statement that encourages healthcare practitioners to view a history of illicit drug use in the treatment of chronic pain as a complicating factor, not a direct contraindication [26]. The study is unique in that it contributes the perspectives of a traditionally “hard-to-reach” population due to their work and threats of criminalization [42]. Finally, this study demonstrates that Sex Workers should not only participate in but should lead development and implementation of research and programs for managing chronic pain in the setting of addiction. This information will be used to guide future community-based research in chronic pain and addictions in female Survival Sex Workers.

## Footnotes

1 The term Aboriginal is used in the Canadian context as a distinction inclusive of First Nations, Metis and Inuit peoples, according to the Royal Commission on Aboriginal Peoples, 1996 [43].
